# Sociodemographic factors associated with HPV awareness/knowledge and cervical cancer screening behaviors among caregivers in the U.S

**DOI:** 10.1186/s12905-022-01918-4

**Published:** 2022-08-08

**Authors:** Jiyeong Kim, Melanie S. Dove, Julie H. T. Dang

**Affiliations:** Division of Health Policy and Management, Department of Public Health Sciences, University of California, Davis, Davis, USA

**Keywords:** Human papillomavirus (HPV), HPV awareness, Cervical cancer screening, Caregivers, Health disparities, Sociodemographic

## Abstract

**Background:**

Family caregivers may be at a higher risk for several chronic diseases, including cancer. Cervical cancer is one of the most common causes of cancer death among women. Despite family caregivers’ vulnerability, the status of their HPV awareness, knowledge, and preventive health behaviors, including cervical cancer screening, has been understudied. Thus, this study aimed to examine the sociodemographic factors associated with HPV awareness and knowledge and adherence to the cervical cancer screening guidelines among caregivers in the U.S.

**Methods:**

Nationally representative cross-sectional survey data were obtained from the Health Information National Trends Survey (HINTS 5, 2017–2020). Female caregivers aged 21–65 were included (N = 1190). Weighted multivariable logistic regression was performed to identify factors associated with HPV awareness (heard of HPV), knowledge (HPV can cause cervical cancer), and adherence to the United States Preventive Service Task Force 2018 cervical cancer screening guidelines by sociodemographic factors (age, race/ethnicity, education, household income, marital status,) and the intensity of caregiving.

**Results:**

An estimated 79% of female caregivers were aware of HPV and 84% adhered to the cervical cancer screening guidelines. Caregivers who were older than 50 (OR = 3.62, 1.91–6.85, adherence of *aged* 21–50 vs. 51–65), Hispanics of race/ethnicity compared with Black/African Americans (OR = 3.14, 1.31–7.52, adherence of *Black/African Americans* vs. *Hispanics*), with a high school education or less (OR = 2.34, 1.14–4.82, adherence of *Some college or more* vs. *High school education or less*), and with intense caregiving duty (spending 35 h/week or more on caregiving) compared with light-duty (OR = 2.34, 1.10–5.00, adherence of 5–14 h vs. 35 h* or more,* weekly) had poor adherence to the cervical cancer screening guidelines. Caregivers who were older, racial minorities (Asian, Native Hawaiian/Pacific Islander, American Indian/Alaska Native, Multiple races), and less educated showed lower HPV awareness (Heard of HPV) than their counterparts.

**Conclusions:**

There are caregiving populations whose HPV awareness and cervical cancer screening adherence are low. To improve their awareness and knowledge of HPV and support their cervical cancer screening behaviors, we need to consider interventions that target those specific populations.

## Introduction

A family caregiver is defined as “any relative, partner, friend or neighbor who has a significant personal relationship with, and provides a broad range of assistance for, an older person or an adult with a chronic or disabling condition” [1]. Approximately 53 million individuals were family caregivers in the U.S. in 2020 [2]. More than one in five people (21%) provided unpaid healthcare or functional needs for their family members [2]. Caregivers are often described as hidden patients because caregiving is burdensome as it requires physical, emotional, and financial sacrifices and is usually a long-term commitment, spanning from several years to over a decade [3]. About a quarter of caregivers (26%) report spending over 20 h per week providing care [4]. Consequentially, this population is vulnerable to an unhealthy lifestyle, including insufficient sleep, and is at higher risk for chronic diseases, including depressive disorder and cancer [5–9].

The majority of caregivers are female (61%) [4], and the average age of female caregivers is 50.1 years [2]. Cervical cancer is the most common gynecological cancer and the average age at diagnosis is 50 [10]. It is also one of the most common causes of cancer death among U.S. women despite advances in cervical cancer prevention and treatment [10]. Over 90% of cervical cancer can be attributable to high-risk Human Papillomavirus infection. Papanicolaou cytology (Pap smear) detects cell changes caused by HPV and allows at-risk women to receive treatment before it becomes invasive carcinoma. As early detection can significantly reduce cervical cancer incidence and mortality, active cervical cancer screening is strongly recommended as an effective prevention strategy. The U.S. Preventive Services Task Force (USPSTF 2018) recommends cervical cytology every 3 years for women aged 21–29 years old and for women aged 30–65 years old, either cervical cytology every 3 years, high-risk Human papillomavirus (hrHPV) testing every 5 years, or hrHPV testing in combination with cytology (co-testing) every 5 years [11]. These guidelines, updated in 2018, highlight the importance of adhering to preventative cervical cancer screenings while providing more options for hrHPV testing in addition to Pap testing.

In 2022, 14,100 new cervical cancer cases and 4280 cervical cancer death were reported despite effective prevention and treatment options [12]. In the latest report, the disparities in cervical cancer incidence and mortality by socioeconomic status among U.S. women were still observed [12]. Despite the threat of cervical cancer, knowledge regarding the causes of cervical cancer and the linkage to HPV infection (referred to as HPV knowledge) has been low to moderate among Americans [13–15]. Furthermore, HPV knowledge level differs by sociodemographic characteristics (e.g., race/ethnicity, age, income, educational attainment, insurance status, rurality of residence) [15–22]. HPV knowledge level was lower in racial/ethnic minorities (particularly among Hmong and Korean Asian Americans and Hispanics), older populations, and rural residents [15–22]. Adherence to the cervical cancer screening guidelines (e.g., pap smear within the past 3 years) has been moderate to high [23, 24]. Previous analysis of HINTS data (2013–2014) revealed that 81.3% of 21–65 years reported that they had a Pap smear in the past 3 years [25]. However, stark disparities in sociodemographic factors were observed in cervical cancer screening behavior [3–5, 23, 26–32]. For example, pap test was less utilized among women who are older, racial/ethnic minorities (African American, Asians, Hispanics) [4, 26, 32], and women with low socioeconomic status (SES). Low SES includes women with low income, low educational attainment, no health insurance, and women who lack a usual source of healthcare [3, 5, 27–31].

While multiple studies have shown disparities in HPV knowledge and cervical cancer screening behavior in women in the U.S., studies focused on family caregivers’ negative health behaviors due to the caregiving burden and the receipt of preventive clinical services, including cancer screenings, are still scant with inconsistent results [33–42]. Studies have reported that caregivers’ were less likely to receive cervical cancer screening because of caregiving duties [33–36], caregivers were more aware of the preventive health services and hence actively participating in them [39–42], and or have reported no association [34, 37, 38].

Despite the inconsistent findings, there has been a longstanding concern that caregivers are less likely to receive cancer screenings because the burden of caregiving may hinder them from obtaining care for themselves. This is alarming as their health status is already vulnerable [5–8, 33–36]. While we can reasonably assume that subgroups of caregivers may have disproportionately low cervical cancer screening rates and low HPV awareness and knowledge, little is known in this area. Knowing which caregiver subgroups are not up to date with their cervical cancer screening and which subgroups lack HPV knowledge could contribute to developing targeted interventions to mitigate existing inequities [43]. Therefore, this study aimed to identify sociodemographic factors associated with disparities in HPV awareness and knowledge and cervical cancer screening behaviors among female caregivers in the U.S.

## Methods

### Data source

This study used publicly available cross-sectional data from the Health Information National Trends Survey (HINTS) [44]. HINTS is a self-administered nationally representative survey conducted by the National Cancer Institute. The present study used HINTS 5 Cycles 1–4 in 2017–2020. HINTS 5 is a single-mode mailed survey using a two-stage sample design except for Cycle 3, as Cycle 3 employed a push-to web pilot method in addition to the mailed survey. HINTS 5 included data on the caregiver population, their health-related behavior, perception, and knowledge of the disease. Geographic addresses were stratified by areas with a high or low concentration of minority populations in HINTS 5 Cycles 2–4. HINTS 5 Cycle 1 included one more stratification in geographic address, counties of Central Appalachia. The present study followed the Strengthening the Reporting of Observational Studies in Epidemiology (STROBE) guideline [45]. HINTS 5 included 16,092 respondents; 3285 in cycle 1 (2017, response rate: 32.4%), 3504 in cycle 2 (2018, response rate: 32.4%), 5,438 in cycle 3 (2019, response rate: 30.3%), and 3865 in cycle 4 (2020, response rate: 36.7%). The response rate was estimated based on the Response Rate 2 formula from the American Association of Public Opinion Research [46]. In our analysis, we included female caregivers aged 21–65 (n = 1190) because that is the population recommended to obtain a regular cervical cancer screening every 3 years by the USPSTF 2018.

### Caregiver status

Caregiving status was identified by one question; “Are you currently caring for or making health care decisions for someone with a medical, behavioral, disability, or other condition?” Those who affirmatively responded ‘yes’ were defined as caregivers.

### Outcome variables

#### HPV awareness (Heard of HPV) and HPV knowledge (HPV can cause cervical cancer)

The following question was used to investigate caregivers’ awareness of Human Papillomavirus (HPV): “Have you ever heard of HPV? HPV stands for Human Papilloma Virus. It is not HCV, HIV, or Herpes.” Among respondents who answered, ‘yes’ to HPV awareness, HPV knowledge was assessed with the question, “Do you think HPV can cause cervical cancer?” In this study, HPV knowledge was defined as having knowledge of the causal effect of HPV on cervical cancer.

#### Adherence to the cervical cancer screening guidelines (Had a pap test within 3 years)

To distinguish the caregivers who were compliant with the USPSTF 2018 cervical cancer screening guidelines, the question, “How long ago did you have your most recent Pap test to check for cervical cancer?” was used. Those who answered, “I have never had a Pap test,” were re-coded to “Never.” Caregivers with all other answers were divided into “within guidelines” if they had a pap test within the past 3 years or “outside guidelines” if they had a pap test more than 3 years ago. As only 18 women reported never receiving a Pap test, we only included ‘within guidelines’ and ‘outside guidelines’ in our analysis, and ‘Never’ was excluded.

### Covariates

The social determinants of health framework from the Healthy People 2030 was used to determine the sociodemographic predictors for this study [47]: Age (21–50, 51–65), race/ethnicity (White, Hispanic, African American/Black, Others including Asian, Native Hawaiian/Pacific Islander, American Indian/Alaska Native, Multiple races), household income (less than $20,000, $20,000 to less than $35,000, $35,000 to less than $50,000, $50,000 or more), educational attainment (high school graduate or less, some college or more), marital status (married or living with a romantic partner as married vs. not married including divorced, widowed, separated, single/never been married), employment status (employed vs. unemployed including homemaker, student, retired, disabled), health insurance type (insured by employment or private insurance, Tricare/VA/Indian Health Services, Medicare, Medicaid). Besides the sociodemographic factors, weekly time spent on caregiving (less than 5 h, 5–14 h, 15–20 h, 21–34 h, 35 h or more) was included.

### Statistical analysis

The prevalence of HPV awareness, HPV knowledge, and adherence to cervical cancer screening guidelines were reported [frequency, weighted percentage, and 95% confidence interval (95% CI)]. To examine the association between characteristics and the three outcomes of interest (1) HPV awareness (heard of HPV), (2) HPV knowledge (HPV can cause cervical cancer), and (3) adherence to the cervical cancer screening guidelines (a Pap smear within 3 years), weighted multivariable logistic regression was performed to obtain odds ratios (ORs) and 95% CIs. Models for HPV awareness and knowledge were adjusted by sociodemographic factors, and the model for screening uptake was additionally adjusted for time spent on caregiving. The statistical significance was determined at a *p* value less than 0.05. A complete case analysis approach was used to manage missing data. All estimates accounted for the sampling strategy using full sample weights and survey procedures in SAS (SAS Studio, version 9.4, Cary, NC).

## Results

Among female caregivers aged 21–65, 39% were aged 51–65, 63% were non-Hispanic White, 46% reported less than $50,000 annual household income, 24% had a high school education or less, 60% were employed, 64% were married, 67% were insured by an employment-based or private plan while 33% were insured by a government health plan (Medicare, Medicaid, Tricare, VA, Indian Health Services), and approximately 98% were urban residents. An estimated 33% spent 35 h or more on caregiving activities weekly (Table 1).

**Table 1:** Demographic characteristics of the female caregiving population 2017–2020 (HINTS 5)

	Frequency, n (N = 1190)	Weighted Percentage, % (95% CI)
*Age (years)*		
21–50	612	61.0 (56.7, 65.4)
51–65	578	39.0 (34.6, 43.3)
*Race/ethnicity*		
Non-Hispanic White	654	63.0 (58.8, 67.2)
Black/African American	197	13.0 (10.5, 15.5)
Hispanic	190	14.7 (12.0, 17.3)
Others^+^	97	9.3 (6.08, 12.6)
*Household income*		
Less than $20,000	210	19.6 (15.9, 23.4)
$20,000-less than $35,000	133	11.7 (9.18, 14.2)
$35,000-less than $50,000	152	14.3 (11.5, 17.0)
$50,000 or more	632	54.4 (50.4, 58.5)
*Years of education*		
High school or less	221	24.1 (20.5, 27.7)
Some college or more	962	75.9 (72.3, 79.5)
Area: rurality		
Urban	1163	97.9 (96.9, 98.8)
Rural	27	2.1 (1.15, 3.07)
*Employment*		
Employed	501	59.5 (54.5, 64.5)
Unemployed	308	40.5 (35.4, 45.6)
*Marital status*		
Married	709	64.5 (60.4, 68.5)
Not married	472	35.5 (31.5, 39.6)
*Health insurance*		
By employment or private	692	67.4 (63.0, 71.7)
Tricare, VA, IHS*	48	4.1 (2.19, 6.07)
Medicare	59	3.9 (2.46, 5.35)
Medicaid	218	24.6 (20.4, 28.8)
*Time spent on caregiving (week)*		
Less than 5 h	244	27.5 (23.4, 31.6)
5 to 14 h	206	21.3 (18.0, 24.8)
15 to 20 h	78	8.9 (5.97, 11.8)
21 to 34 h	57	8.9 (5.56, 12.2)
35 h or more	241	33.4 (28.5, 38.3)

*IHS: Indian Health Services; ^+^Others: Asian, Native Hawaiian/Pacific Islander, American Indian/Alaska Native, Multiple races

### HPV awareness (Heard of HPV) and HPV knowledge (HPV can cause cervical cancer)

Approximately 79% of female caregivers (aged 21–65) heard of HPV, and the majority of participants thought that HPV could cause cervical cancer (99%) (Table 2). However, disparities in HPV awareness were found by sociodemographic characteristics (Table 3). Younger female caregivers (21–50) were nearly 2.5 times as likely to have heard of HPV compared with older caregivers [50–64] (OR = 2.47, 95% CI = 1.49, 4.08). Hispanic caregivers had 3.5 times the odds of having heard of HPV compared to other race/ethnic groups (OR = 0.29, 95% CI = 0.12, 0.70). Female caregivers with some college or more education had 3 times the odds of having heard of HPV compared with their less-educated counterparts (OR = 2.92, 95% CI = 1.72, 4.94).

**Table 2:** Caregiver's HPV awareness/knowledge and cervical cancer screening behaviors in the U.S. (N, %)

	HPV Awareness		HPV Knowledge*		Pap Test in 3 Years**	
	N/row total	Weighted percent % (95% CI)	N/row total	Weighted percent % (95% CI)	N/row total	Weighted percent % (95% CI)
Total	961/1189	79%	803/818	99%	968/1152	84%
*Age (years)*						
21–50	527/611	84 (80, 88)	460/467	99 (97, 100)	534/592	88 (84, 91)
51–65	434/578	70 (63, 76)	343/351	98 (97, 100)	434/560	77 (72, 82)
*Race/ethnicity*						
White	572/653	86 (82, 90)	487/493	99 (98, 100)	538/641	84 (80, 88)
Black/African Amer	148/197	71 (61, 80)	116/118	99 (97, 100)	161/188	88 (81, 95)
Hispanic	148/190	79 (72, 86)	124/126	99 (97, 100)	147/182	77 (69, 84)
Others^+^	66/97	54 (37, 70)	57/59	95 (86, 100)	79/91	84 (72, 97)
*Household income*						
Less than $20,000	149/209	73 (63, 83)	112/118	98 (96, 100)	153/199	78 (69, 86)
$20,000 to < $35,000	97/133	72 (62, 82)	72/75	96 (89, 100)	97/128	82 (75, 90)
$35,000 to < $50,000	119/152	80 (71, 88)	88/90	97 (92, 100)	123/148	84 (77, 91)
$50,000 or more	552/632	82 (77, 88)	494/498	99 (98, 100)	546/618	86 (82, 90)
*Years of education*						
High school or less	140/221	65 (56, 73)	94/101	97 (95, 100)	158/208	75 (66, 83)
Some college or more	817/961	83 (80, 87)	705/713	99 (98, 100)	806/939	86 (83, 89)
*Employment*						
Employed	407/501	78 (72, 85)	348/354	98 (96, 100)	410/484	85 (81, 90)
Unemployed	234/307	74 (67, 81)	185/192	98 (96, 100)	241/298	83 (77, 88)
*Marital status*						
Married	595/709	82 (78, 87)	511/518	98 (97, 100)	590/690	83 (80, 87)
Not married	360/471	73 (66, 80)	287/295	99 (98, 100)	369/453	83 (78, 88)
*Health insurance*						
Employ/private	592/692	82 (77, 88)	518/512	99 (99, 100)	595/675	88 (84, 91)
Tricare/VA/IHS*	40/48	86 (74, 97)	35/36	99 (97, 100)	39/47	80 (59, 100)
Medicare	45/58	70 (51, 89)	32/34	90 (73, 100)	42/55	87 (76, 98)
Medicaid	166/218	75 (67, 84)	130/135	97 (94, 100)	177/212	83 (76, 89)

*IHS: Indian Health Services

^+^Others: Asian, Native Hawaiian/Pacific Islander, American Indian/Alaska Native, Multiple races

*Among those with HPV awareness

**Among those who ever had a Pap test

**Table 3:** Caregivers’ HPV awareness and knowledge in the U.S. (ORs, 95% CIs)

	HPV awareness (n = 1189) (Heard of HPV) Odds ratio (95% CI)	HPV knowledge** (n = 818) (HPV can cause Cervical Cancer) Odds ratio (95% CI)
*Age (Years)*		
21–50	2.47 (1.49, 4.08)*	1.22 (0.27, 5.42)
51–65	Reference	Reference
*Race/ethnicity*		
White	1.83 (0.96, 3.49)	0.88 (0.12, 6.38)
Black/African American	0.81 (0.39, 1.68)	0.65 (0.06, 7.45)
Hispanic	Reference	Reference
Others^+^	0.29 (0.12, 0.70)*	0.17 (0.01, 2.05)
*Household income*		
Less than $20,000	Reference	Reference
$20,000-less than $35,000	0.70 (0.30, 1.61)	0.26 (0.04, 1.80)
$35,000-less than $50,000	1.05 (0.48, 2.26)	0.39 (0.07, 2.28)
$50,000 or more	1.08 (0.54, 2.16)	2.65 (0.44, 16.08)
*Years of education*		
High school or less	Reference	Reference
Some college or more	2.92 (1.72, 4.94)*	1.46 (0.31, 6.74)
*Marital status*		
Married	1.43 (0.84, 2.43)	0.30 (0.05, 1.64)
Not married	Reference	Reference

*Bold-coded is statistically significant, *p* value < 0.05

^+^Others: Asian, Native Hawaiian/Pacific Islander, American Indian/Alaska Native, Multiple races

**Among those with HPV awareness

### Adherence to the cervical cancer screening guidelines (Had a pap test within 3 years)

Among female caregivers who had ever had a pap test, 84% had a pap test in the past 3 years (Table 2). As shown in Table 4, younger caregivers (21–50 years) were 3.5 times as likely to follow the cervical cancer screening guidelines compared with older caregivers (51–65 years) (OR = 3.62, 95% CI = 1.91, 6.85). Black/African American caregivers were 3 times as likely to adhere to the cervical cancer screening guidelines compared with Hispanics (OR = 3.14, 95% CI = 1.31, 7.52). More educated caregivers (some college or more) were almost 2.5 times as likely to obtain cervical cancer screening compared with their less-educated counterparts (high school or less) (OR = 2.34, 95% CI = 1.14, 4.82). Caregivers who spent fewer hours on caregiving (5–14 h weekly) were almost 2.5 times as likely to adhere to the screening guidelines as those who spent most hours (35 h or more) (OR = 2.34, 95% CI = 1.10, 5.00).

**Table 4:** Caregivers’ adherence to cervical cancer screening guidelines in the U.S. (ORs, 95% CIs)

	Adherence to the Guidelines** (N = 1152) (Last Pap smear in 3 years) Odds ratio (95% CI)
*Age (Years)*	
21–50	3.62 (1.91, 6.85)*
51–65	Reference
*Race/ethnicity*	
White	1.88 (0.98, 3.60)
Black/African American	3.14 (1.31, 7.52)*
Hispanic	Reference
Others^+^	0.84 (0.25, 2.81)
*Years of education*	
High school or less	Reference
Some college or more	2.34 (1.14, 4.82)*
*Time spent on caregiving (week)*	
Less than 5 h	1.77 (0.79, 3.95)
5–14 h	2.34 (1.10, 5.00)*
15–20 h	1.07 (0.36, 3.19)
21–34 h	1.28 (0.47, 3.52)
35 h or more	Reference

*Bold-coded is statistically significant, *p* value < 0.05

^+^Others: Asian, Native Hawaiian/Pacific Islander, American Indian/Alaska Native, Multiple races

**Among those who ever had a Pap test

## Discussion

This study assessed sociodemographic factors associated with HPV awareness (heard of HPV), HPV knowledge (HPV can cause cervical cancer), and adherence to the cervical cancer screening guidelines (had a pap test within 3 years) among female caregivers in the U.S. from 2017 to 2020, using nationally representative survey data (HINTS 5, Cycles 1–4). Female caregivers who were older, racial/ethnic minorities, and those with less education had lower HPV awareness and cervical cancer screening adherence. Intense caregiving duty (spending 35 h/week or more on caregiving) was also associated with low adherence to the cervical cancer screening guidelines. Compared to the general population in the U.S., there were some similarities and differences. Similar to women in the general population, women caregivers who were older, racial/ethnic minorities, and those who were less educated were less likely to be aware of HPV and less likely to adhere to the cervical cancer screening guidelines [48–51]. However, income was associated with HPV awareness in the general population, but not among caregivers, and age and education were not related to HPV knowledge among caregivers, unlike the general U.S. population [48].

Older age was associated with low HPV awareness and low uptake of cervical cancer screening among caregivers. Plausible explanations could include that HPV vaccination campaigns and recommendations target young girls and/or healthcare professionals may focus more on younger age groups (11–12 years rather than 21–26 years) to promote HPV vaccine uptake and series completion [52–54]. This could result in more chances of being exposed to the HPV awareness campaigns among young teenagers and their mothers, who are most likely in their 30 s or 40 s. Older caregivers were far behind (75%) the targeted adherence to the cervical cancer screening guidelines (84.3%) set by Healthy People 2030 among U.S. women [55] compared to their younger counterparts (91%). The association between age and adherence to the screening guidelines could be explained by the relationship that low HPV awareness and knowledge are closely related to low cervical cancer screening [56, 57]. Considering the average diagnosed age of cervical cancer in the U.S. is 50, efforts targeting this older age group are needed to improve and maintain their adherence to the USPSTF guidelines.

We found that HPV awareness was lower among caregivers who were racial/ethnic minorities. Specifically, Hispanic caregivers were less likely to obtain preventive cancer screening behaviors compared with their Black/African American counterparts. Low HPV awareness and knowledge and low cervical cancer screening uptake has been previously reported among Hmong, a growing Southeast Asian American population, and Korean Asian Americans, and Hispanic women [18, 19, 21, 22, 50, 51, 58, 59].

We also found that HPV awareness was lower among caregivers who had less educational achievement. Individuals with higher education and higher income are more likely to have higher health literacy and greater access to health resources, which can possibly increase their knowledge and awareness of HPV. However, HPV knowledge was not different by socioeconomic status in our study. A potential explanation could be that only those who were already aware of HPV were asked further HPV knowledge questions. Thus, it would have been challenging to identify sociodemographic disparities in HPV knowledge in our sample. Our findings support the need for tailored interventions that take into consideration the individual (e.g., lack of knowledge in cervical cancer etiology and screening services available) [59, 60], sociocultural (e.g., low levels of acculturation, gynecologic clinic attending is hindered by male partner) [61, 62], and systematic barriers (e.g., lack of access to the services) [63, 64] to cervical cancer screening services among Hispanic caregivers.

Long caregiving hours (35 h or more weekly) were associated with low adherence to cervical cancer screening guidelines among caregivers. Several studies that examined the associations between caregiving, the caregiving burden, and low engagement in preventative cancer services reported inconclusive results [36–38, 65]. Our findings provide evidence to strengthen the rationale for developing tailored strategies targeting individuals with intense caregiving duty to promote cancer screening behaviors. Further studies will be warranted to comprehensively assess the associations of caregiving burden with cancer screening uptake, using standardized measures (e.g., Zarit score) with larger sample sizes. Additionally, a qualitative approach would be warranted to examine the detailed barriers that hinder cervical cancer screening uptake for those with poor adherence.

A recent study reported that the most common reason for not receiving timely screening was lack of screening knowledge [66]. Addressing disparities in HPV awareness and knowledge and cervical cancer screening behaviors is important for caregivers because this population needs additional support to promote their optimal health given their at-risk health condition and crucial role in care recipients’ health [67, 68]. Determining which caregiver subgroups are overdue for cervical cancer screenings could inform relevant stakeholders, including clinicians, who have a primary role in recommending screening to eligible caregivers [66].

### Limitation

The current study has several limitations. First, HINTS is a cross-sectional survey and cannot provide knowledge of temporality or causal associations. Second, as HINTS data is self-reported, we need to acknowledge the possibility of respondent biases. Respondents may have mis-reported the dates of their last Pap Smear test, especially if it happened 2–3 years ago (recall bias). Moreover, respondents might not always honestly report their knowledge and behaviors, which potentially could lead to either an increase or decrease in screening rates (reporting bias). Third, the small sample size may be related to some of the non-significant results. Fourth, there is a possibility of mislabeling the screening guideline-compliant individuals as non-compliant due to the potential confusion between hrHPV test and a Pap smear. As HINTS only asks about a Pap smear as a preventative screening service for cervical cancer, we might have underestimated the percent who were screening-compliant, as we did not include those who received hrHPV testing every 5 years. Since there is an increasing trend of using hrHPV test over a Pap smear due to convenience in recent years [69], we would suggest HINTS incorporate hrHPV testing into the Pap smear questionnaire to properly specify people with preventative cervical cancer screening behaviors. Fifth, potentially, the changes in cervical cancer screening guidelines that happened over the survey period might have affected women's cervical cancer screening knowledge and behaviors [66]. Last, this study did not include the rurality of residence as a sociodemographic factor despite the potential association with disparities in HPV awareness and knowledge as well as adherence to the guidelines of cervical cancer screening [67, 69, 70]. Because nearly 98% of caregivers were urban residents, we did not think we could have meaningful results out of this population distribution. However, further investigation is needed to better understand how rurality of residence is associated with caregivers' cervical cancer screening behaviors, including the caregiving burden among rural residents [70].

## Conclusion

We found that sociodemographic factors, particularly age, race/ethnicity, educational attainment, and the intensity of caregiving, were associated with disparities in HPV awareness and adherence to the cervical cancer screening guidelines among female caregivers in the U.S. The value of this study is the suggestion that targeted interventions should focus on female caregivers who are aged 51–65, racial/ethnic minorities (Hispanics and other race/ethnic groups, including Asian, Native Hawaiian/Pacific Islander, American Indian/Alaska Native, Multiple races), have low educational attainment (high school graduate or less) and spend 35 h or more on caregiving weekly. Therefore, targeted interventions to improve HPV awareness and cervical cancer screening adherence are urgently required to mitigate existing inequities among these vulnerable subgroups of caregiving women in the U.S.

## Data Availability

The datasets generated and/or analyzed during the current study are available in the Health Information National Trends Survey, https://hints.cancer.gov/data/default.aspx

## Abbreviations

HINTS: Health Information National Trends Survey
HPV: Human papillomavirus
BRFSS: Behavioral Risk Factor Surveillance System
Pap Smear: Papanicolaou cytology
USPSTF: U.S. Preventive Services Task Force
